# Response of atmospheric deposition and surface water chemistry to the COVID-19 lockdown in an alpine area

**DOI:** 10.1007/s11356-022-20080-w

**Published:** 2022-04-09

**Authors:** Michela Rogora, Sandra Steingruber, Aldo Marchetto, Rosario Mosello, Paola Giacomotti, Arianna Orru’, Gabriele A. Tartari, Rocco Tiberti

**Affiliations:** 1 National Research Council of Italy, Water Research Institute (CNR-IRSA) , Largo Tonolli 50, 28922 Verbania Pallanza (VB), Italy; 2 Ufficio dell’Aria, del Clima e e delle Energie Rinnovabili, Dipartimento del Territorio del Cantone Ticino, CH-6501 Bellinzona, Switzerland; 3 Department of Earth and Environmental Sciences DSTA, University of Pavia, Via Ferrata 9, 27100 Pavia, Italy

**Keywords:** Air pollution, Nitrate, Emission reduction, Mountain lakes, Long-term data

## Abstract

The effects of the COVID-19 lockdown on deposition and surface water chemistry were investigated in an area south of the Alps. Long-term data provided by the monitoring networks revealed that the deposition of sulfur and nitrogen compounds in this area has stabilized since around 2010; in 2020, however, both concentrations and deposition were significantly below the average values of the previous decade for SO_4_ and NO_3_. Less evident changes were observed for NH_4_ and base cation. The estimated decrease of deposition in 2020 with respect to the previous decade was on average − 54% and − 46% for SO_4_ and NO_3_, respectively. The lower deposition of SO_4_ and NO_3_ recorded in 2020 was caused by the sharp decrease of SO_2_ and particularly of NO_x_ air concentrations mainly due to the mobility restrictions consequent to the COVID-19 lockdown. The limited effects on NH_4_ deposition can be explained by the fact that NH_3_ emission was not affected by the lockdown, being mainly related to agricultural activities. A widespread response to the decreased deposition of S and N compounds was observed in a group of pristine freshwater sites, with NO_3_ concentrations in 2020 clearly below the long-term average. The rapid chemical recovery observed at freshwater sites in response to the sharp decrease of deposition put in evidence the high resilience potential of freshwater ecosystems in pristine regions and demonstrated the great potential of emission reduction policy in producing further substantial ameliorations of the water quality at sensitive sites.

**Supplementary Information:**

The online version contains supplementary material available at 10.1007/s11356-022-20080-w.

## Introduction

The lockdown imposed by many countries all over the world to control the spread of COVID-19 provided an insight into the effects of reduced human mobility on natural ecosystems. Rutz et al. (2020) defined the global slowing of modern human activities during the lockdown as “anthropause”: this transient but unprecedented condition fostered important changes in natural ecosystems and modified the interaction between humans and environment. Positive effects of the “anthropause” on biodiversity and on the quality of specific environmental compartments have been reported (Manenti et al. 2020; Schrimpf et al. 2021; Patterson et al. 2021). In particular, the effects of the lockdown were notable on air quality, as transport and mobility in general are the major contributors to air pollutant emissions.

Numerous studies, based on both measured and modelled data, analyzed the effects of lockdown on air quality, both at regional/national (Menut et al. 2020; Wyche et al. 2021) and global levels (Habibi et al. 2020; Shi et al. 2021; Venter et al. 2020). Studies considered different air pollutants, including PM2.5, PM10, NO_x_, SO_x_, NH_3_, and ozone. In most cases, these studies highlighted the prominent effects that lockdown measures had on NO_x_ concentrations with respect to other pollutants (Liu et al. 2021; Ciarelli et al. 2021).

Italy was the first country in Europe to enforce lockdown measures aimed at reducing the spread of COVID-19 (Ciarelli et al. 2021). The first lockdowns were enforced in Northern Italy (Lombardy and Veneto regions) in February 2020 and soon after in the other regions until the national lockdown on 9 March 2020. In southern Switzerland, Canton Ticino was the first region in the country to introduce restrictions, because this area experienced an earlier increase in infections due to the proximity to the northern Italian outbreak (Grange et al. 2020). As a consequence of lockdown measures, mobility was largely reduced, and a drop in vehicle traffic occurred. Many productions and industrial activities were also locked down from March, except for agricultural ones (Lovarelli et al. 2020). The restrictions lasted until the end of May, and then most of the activities gradually restarted. However, some restrictions were maintained at local or regional level for the whole year 2020, particularly during the so-called second wave of autumn 2020: in Italy, schools only partially reopened, smart working was implemented both in the public and private sectors, and mobility between different cities and regions was restricted according to the average regional evolution of the epidemic, contributing to reduction of road traffic and related emissions.

Guevara et al. (2021), using Copernicus data, estimated that during the most severe lockdown period, the average emission reductions were − 33% for NO_x_, − 7% for SOx_x_, and − 7% for PM2.5 at the EU-30 level, with even higher reductions in countries where the lockdown restrictions were more severe such as Italy (e.g., − 50% NO_x_, − 12% SO_x_). In a study focused on major cities in Central Italy, Donzelli et al. (2020) observed a significant decrease in NO_2_ concentrations at air quality monitoring stations. Similarly, Lovarelli et al. (2020) reported a decrease of NO_x_ emissions in March 2020 with respect to the average values for 2016–2019 in some provinces of the Lombardy region, Italy. Ciarelli et al. (2021) estimated that lockdown measures reduced NO_2_ air concentrations by up to 46% and 25% in the Po Valley and Swiss Plateau regions, respectively. Putaud et al. (2021) showed that NO_2_ air concentrations decreased because of the lockdown by − 30% and − 40% on average at urban and regional background in Northern Italy. In Switzerland, the analysis of air quality data indicated that NO_2_ and NO_x_ concentrations had decreased in most locations by up to 44 and 58%, respectively, due to the lockdown measures (Grange et al. 2020). The LIFE project PREPAIR (www.lifeprepair.eu) focused on the Po basin area in Northern Italy, providing a detailed analysis of the effects of lockdown measures on pollutant emissions and air quality: the air concentrations of traffic-related pollutants (NO_2_, NO, benzene) dropped between February and March, while NH_3_ concentrations remained fairly stable (Deserti et al. 2020).

A few studies also considered the effects of COVID-19 lockdown on water quality, mainly using satellite data (Yunus et al. 2020; Tokatlı and Varol 2021). These studies mostly focused on highly impacted ecosystems and on water quality indicators such as suspended particulate matter (Yunus et al. 2020), water transparency and/or turbidity, and chlorophyll concentration (Braga et al. 2020; Shafeeque et al. 2021). These studies showed a general improvement of water quality which was related to the reduction of different anthropogenic pressures such as boat traffic and tourism, wastewater inputs, and industrial effluents (Patterson et al. 2021).

Atmospheric deposition is an important vehicle of chemical compounds to terrestrial and aquatic ecosystems, especially in remote areas and in nutrient-poor habitats (Schindler 1988; Keene et al. 2015; Lepori and Keck 2012) where deposition may be the main — if not the only — source of nutrients (phosphorus and nitrogen compounds) but also a vehicle of atmospheric pollutants (acidifying compounds, POPs, heavy metals) transported with the air masses from source regions (Carrera et al. 2002; Driscoll et al. 2003). Nitrogen deposition has received particular attention due to the important effects that nitrogen may have on ecosystems both as an acidifying and eutrophying agent (Fenn et al. 2003; Elser et al. 2009).

Monitoring networks of atmospheric deposition has been established since the late 1970s at both national and international levels, with the aim to quantify chemical loads, assess long-term trends, and relate atmospheric inputs with critical loads for target ecosystems: examples are the monitoring sites within the ICP FOREST network in Europe (http://icp-forests.net/; Waldner et al. 2014) and the National Atmospheric Deposition Program (NADP) in the USA (http://nadp.slh.wisc.edu/). A contribution to the assessment of atmospheric fluxes of acidifying and eutrophying compounds, photochemical oxidants, and particulate matter also came from modelling work such as those performed under the co-operative programme for monitoring and evaluation of the long-range transmission of air pollutants in Europe (EMEP) (https://www.emep.int/; Simpson et al. 2012).

The deposition of acidity and acidifying agents such as NO_3_ and SO_4_ has caused widespread acidification of sensitive surface water bodies in the 1970s and 1980s; then, extensive recovery has been documented, due to the emission reduction of S and N compounds promoted by international protocols such as the Gothenburg Protocol and its revisions (Stoddard et al. 1999; Skjelkvåle et al. 2005) implemented within the EU Directive 2001/81/EC (European Parliament and Council 2001). The Clean Air Act in the USA and the Air Convention in Europe provided frameworks for international cooperation with the general aim to reduce the negative effects of airborne pollutants. The International Cooperative Programme on Assessment and Monitoring Effects of Air Pollution on Rivers and Lakes (ICP Waters), in particular, was established in 1985, with the specific objective of assessing the degree and extent of atmospheric pollution effects on surface waters (www.icpwaters.no; Kvaeven et al. 2001). ICP Waters monitoring sites have provided evidence of the negative effects of airborne pollutants on freshwaters but also of the positive response of these ecosystems to decreasing emissions (Skjelkvåle et al. 2005; Garmo et al. 2014). Despite the documented benefits obtained with the application of international agreements, atmospheric deposition, especially of N compounds, is still high in some areas to impact vulnerable ecosystems (Baron et al. 2011; Kaste et al. 2020; Bowman et al. 2018). A further reduction of N emissions is needed, especially of ammonia (NH_3_), which decreased much less than N (NO_x_) and S (SO_2_) oxides (Li et al. 2016; Jonson et al. 2021). Recently, the National Emission Ceilings Directive (NECD) (European Parliament and Council, 2016) set 2020 and 2030 emission reduction commitments for the main air pollutants, but it also introduced in Art. 9 the requirement to member states to ensure the monitoring of negative impacts of air pollution upon ecosystems.

Nitrogen deposition represents a fundamental vehicle of N to freshwater, especially to oligotrophic systems (Elser et al. 2009; Bergström and Jansson 2006; Baron et al. 2011). In remote or pristine areas, where direct pollutant sources are absent, water quality of freshwater ecosystems is strongly dependent on atmospheric inputs (Kopáček et al. 1995; Driscoll et al. 2003) which could have important effects such as acidification and nutrient imbalance (Lepori and Keck 2012). These ecosystems may have benefited from the emission reduction consequent to the lockdown, especially as regards NOx. To our knowledge, no studies have analyzed the effects of air emission reduction consequent due to the lockdown on the chemistry of atmospheric deposition and freshwater ecosystems located in remote or scarcely anthropized areas, where deposition represents the main source of pollutants.

We hypothesized that (1) COVID-19 lockdown affected atmospheric deposition and in particular the concentration and deposition of S and N compounds in precipitation because of emission reduction and (2) sensitive freshwater sites responded to these changes. To test these hypotheses, we used deposition and surface water chemistry data collected during the spring lockdown (March to May 2020) and in the following months in Northern Italy/Southern Switzerland, an area which has proved to be highly affected by deposition of atmospheric pollutants.

Despite the restrictions imposed during the lockdown, both in Italy and Switzerland, we were able to maintain the weekly collection and analysis of precipitation samples. Data collected in 2020 were compared with data available from the previous years. We compared the weekly, monthly, and yearly concentrations during the different lockdown phases and the annual deposition of S and N in 2020 with the values recorded in the same periods during the previous decade (2010–2019). We also evaluated the concentrations of SO_4_ and NO_3_ in 2020 at selected freshwater sites in the study area and compared them with previous data. These analyses altogether aimed to assess the recovery potential of sensitive freshwater ecosystems with respect to a transient but relevant reduction of S and N emission consequent to the COVID-19 lockdown.

## Materials and methods

### The monitoring network

**Fig. 1:**
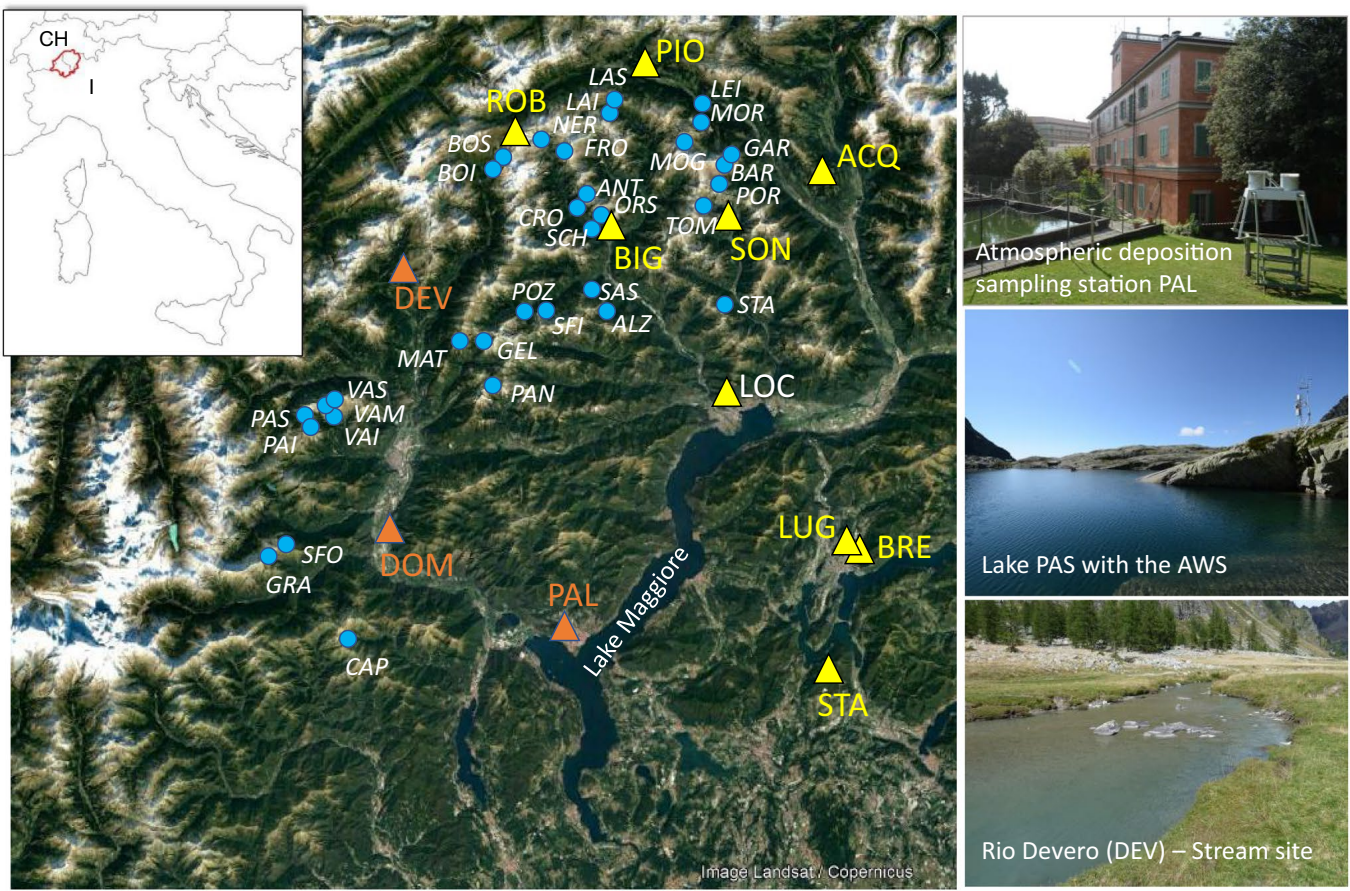
The study area (Piedmont, Italy, and Canton Ticino, Switzerland) with the location of the atmospheric deposition (triangles) and freshwater monitoring sites (circles) and some examples of the study sites (on the right) included in the present study. For the acronyms see Table 1 and Table 1S

The Lake Maggiore watershed (Northern Italy, Southern Switzerland) hosts a large network of sampling stations and sites for the study of atmospheric deposition chemistry and of its effects on the chemistry and biology of recipient freshwater ecosystems (Fig. 1). The network presently includes 12 atmospheric deposition monitoring sites, some subalpine rivers and lakes, and more than 40 high-altitude lakes (above 2000 m a.s.l.; Rogora et al. 2012; 2013). This network was gradually established between the late 1970s and the early 1980s within a consolidated collaboration between Swiss and Italian research institutions both interested in the effects of acidification and nitrogen deposition on terrestrial and aquatic ecosystems. Freshwater sites have contributed data to ICP WATERS since the 1980s (Mosello et al. 2000). Some sites also belong to LTER Italy and LTER Europe (www.lter-europe.net) networks. Some of the Italian freshwater sites have been also included in the recently established network for the monitoring of atmospheric pollution effects on ecosystems in the framework of the NEC Directive (De Marco et al. 2019).

The monitoring network lies in an Alpine area with high rain and snow falls, which are associated to singularly high deposition of pollutants (Rogora et al. 2016). Such pollutants mainly come from the Po Valley, one of the most densely populated and urbanized areas in the world, as well as a hot-spot of atmospheric nitrogen dioxide (NO_2_), which stagnates in the region because the Alps hinders atmospheric circulation (Masetti et al. 2015). Compared to surrounding regions, the precipitation amount in Lake Maggiore area is relatively high due to the orographic effect of the Alps and with a high spatial variability (Saidi et al. 2013).

As an effect of the enforcement of international protocols and the consequent decrease of emissions, a substantial reduction in sulfate and acidity deposition has occurred in the study area, as in most of Europe (Waldner et al., 2014). Deposition of N compounds also decreased since 2000 but at a lesser extent than SO_4_; in particular, NH_4_ decreased the least, progressively accounting for larger proportions of nitrogen deposition, especially at the southern sites, more exposed to pollution (Rogora et al. 2016). Nevertheless, several acid-sensitive sites in the study area recovered from acidification because of decreased acid inputs. The situation also improved as regards the effect of nutrient N: decreasing trends of NO_3_ were indeed observed in several rivers and lakes which were previously affected by N saturation (Rogora et al. 2012). However, very sensitive sites, such as some high-altitude lakes with very low buffer capacity, may still be affected by acidic inputs, especially at snowmelt. Here, nitrates are the main acidifying agent, and despite the recent reduction, N deposition can still affect water chemistry and ecosystems (Rogora et al. 2013; 2016).

### Sampling and analytical methods

The sampling stations for atmospheric deposition are located along a latitudinal and elevational gradient, between 200 and 1900 m a.s.l. (Fig. 1; Table 1). They are all equipped with wet-only collectors. Sampling frequency is weekly, except at one station where samples are collected after each precipitation event. We considered 12 stations (3 in Italy and 9 in Switzerland), which cover the period between 2001 and 2020) (Table 1, Fig. 1).

**Table 1:** Wet-only atmospheric deposition sampling sites in the Italian and Swiss part of Lake Maggiore watershed

Station	Acronym	Country	Latitude	Longitude	Altitude (m a.s.l.)	Activity period
Acquarossa	ACQ	CH	46°25.029′	8°56.018′	575	1990 to present
Bignasco	BIG	CH	46°21.183′	8°36.683′	443	2001 to present
Monte Bre’	BRE	CH	46°0.533′	8°59.283′	925	1995 to present
Locarno	LOC	CH	46°10.650′	8°47.680′	367	1982 to present
Lugano	LUG	CH	46°0.649′	8°56.681′	350	1982 to present
Piotta	PIO	CH	46°31.052′	8°40.993′	1007	1990 to present
Robiei	ROB	CH	46°26.716′	8°30.849′	1890	1996 to present
Sonogno	SON	CH	46°21.083′	8°47.233′	918	2001 to present
Stabio	STA	CH	45°51.117′	8°56.136′	353	1990 to present
Pallanza	PAL	I	45°55.651′	8°34.679′	208	1984 to present
Domodossola	DOM	I	46°7.206′	8°17.915′	270	1986 to present
Devero	DEV	I	46°19.276′	8°16.354′	1634	1996 to present

Within the same collaborative framework, some local and regional Italian and Swiss authorities continued the long-term chemical monitoring of ICP WATERS sites. In the present study, we focus on a subset of ICP WATERS sites characterized by very low levels of anthropogenic disturbance: (i) Rio Devero, an alpine stream with forested catchment, located in the Alpe Veglia and Alpe Devero Natural Park at about 1600 m a.s.l., at the site where one of the deposition monitoring station (DEV) (Table 1) is placed; and (ii) 33 high-altitude lakes (13 in Ossola valley, Italy, and 20 in Canton Ticino, Switzerland) placed above 2000 m a.s.l., ice-covered for most of the year, far away from lowland populated areas and mainly surrounded by sparse vegetation, bare rocks, and, in a few cases, glaciers and rock glaciers (Fig. 1). Both the stream and the lakes were included in previous long-term sampling programs to monitor the effect of atmospheric deposition on freshwaters (acidification and N enrichment): they present varying degrees of sensitivity to acidification (alkalinity between 0 and 800 µeq L^−1^, depending on the catchment lithology) and of NO_3_ concentration (10–30 µeq L^−1^).

The stream site was sampled on a weekly basis since 2004, from the streamside. Lakes in Italy were sampled once per year, at least since 2010 at the end of the ice-free season (usually within 15th September to 15th October). The sampling dates were adapted to avoid sampling during or after heavy precipitation events, which may affect water chemistry. Over the same period (2010–2020), the Swiss lakes were sampled at least twice per year in the late ice-free period; here, we considered the average values of the two samplings. All lake samples were collected at the deepest point of the lake or at the outlet. A focus on two lakes, belonging to the LTER Europe and ICP WATERS networks (Lake Paione Inferiore (PAI) — LTER_EU_IT_088, and Lake Paione Superiore (PAS); LTER_EU_IT_089), is provided. A list of the survey lakes with their main characteristics is provided in Supplementary Material (Table 1S).

Both the samples collected at the atmospheric deposition and freshwater monitoring sites were routinely analyzed for pH, conductivity, alkalinity, ammonium, main cations (calcium, magnesium, sodium, potassium), and anions (sulfate, nitrate, chloride). Base cations (BC) are calculated as the sum of Ca, Mg, Na, and K, and acid-neutralizing capacity (ANC) is obtained as BC less the sum of acid anions (sulfate, nitrate, chloride). The samples were filtered on 0.45-μm filters and analyzed at the Water Research Institute — CNR IRSA (Verbania, Italy) and at the Territory Department of Canton Ticino (Bellinzona, Switzerland). Both laboratories used the same standard methods for freshwater samples (APHA AWWA WEF 2012). The analytical quality and consistency of the data provided by the two laboratories were controlled calculating the ionic balances and by regular intercomparison exercises on rain and surface water analyses (e.g., Escudero-Oñate 2018). For the precipitation samples, the monthly and yearly mean concentrations in precipitations were calculated by weighting weekly concentrations by the sample volumes. Monthly and yearly deposition values were obtained by multiplying such monthly and yearly means by the precipitation amount over the same period.

### Data analysis

Precipitation amount and annual deposition of the main chemical compounds calculated for 2020 were considered together with long-term data available for the period 1996–2019. In the decade 2010–2019, SO_4_, NO_3_, NH_4_, and BC concentrations in precipitation stabilized following a previous decline (Rogora et al. 2016; Steingruber 2018; and unpublished data), we therefore used 2010–2019 as a reference period for the comparison with the lockdown affected period. Then, we compared the monthly concentrations of SO_4_, NO_3_, NH_4_, and BC before (Jan 2010- Feb 2020) and after (until the end of 2020) the COVID-19 lockdown in the atmospheric deposition and lake water samples. We used some linear mixed effects models (LMEs) (Zuur et al. 2009) fitted by maximum likelihood to account for repeated measures at the same sites. LMEs were implemented in the “lme4” package of the statistical environment R version 4.1.1 (R Core Team 2021).

To compare SO_4_, NO_3_, NH_4_, and BC concentrations in precipitation, their monthly concentrations were log-transformed — to approximate normality — and added to the LMEs as dependent variables, with the ID of each sampling station, and the year and the month of sampling as additive random effects. We added a binary factor (COVID-19) encoding for pre- (January 2010 to February 2020) and post-COVID-19 lockdown samplings as a covariate. Since ions are usually more diluted in abundant precipitation, we added the precipitation volume (in mm) and its interaction with COVID-19 as further explicatory variables.

Since the predicted changes in precipitation chemistry are expected to reduce the deposition of SO_4_, NO_3_, NH_4_, and BC and their concentration in natural aquatic habitats, we compared SO_4_, NO_3_, NH_4_, and BC deposition before and after the COVID-19 lockdown, running three more LMEs with the monthly deposition of SO_4_, NO_3_, NH_4_, and BC (log + 1 transformed to approximate normality) as dependent variables, COVID-19 as covariate, and the same random structure described above. We ran two more LMEs to compare the concentrations of SO_4_ and NO_3_ (i.e., the most relevant acidifying compounds) before and after the COVID-19 in the 33 high mountain lakes. In the latter LMEs, we added as dependent variables the log-transformed concentrations of the annual measures of SO_4_ and NO_3_ in the lakes. The lake ID and the year of sampling were added as additive random effects. Since high mountain lakes are natural systems, characterized by a certain heterogeneity, other than the COVID-19 binary factor, we added as covariates some relevant environmental variables which may influence lake hydrochemistry:

- Elevation (scaled around the average value in m a.s.l.) as a proxy of the microclimatic conditions at the sampling sites (Tiberti et al. 2019)
- Lake area (A; in ha) and catchment area–lake area ratio (BA) which is related to weathering rates and consider the sensitivity of lakes towards external factors (e.g., air temperature, water residence times) (Camarero et al. 2009)
- Vegetation cover in the lake catchments (vegetation; as a percentage) which is related to retention processes and nutrient uptake (Marchetto et al. 1995)
- The presence/absence of cryosphere elements — both glaciers and rock glaciers — in the catchment, which may affect water chemistry and pollutant concentrations (Slemmons et al. 2013) and mask the short-term effects of any variation in pollutant deposition
- The presence/absence of sulfur-bearing rocks in the catchment and its interaction with COVID-19 (only for the model concerning [SO_4_]), because the weathering of such rocks may produce larger inputs of SO_4_ and mask the effects of changing deposition (Rogora et al. 2013)

By way of example, we provided a graphic representation of the long-term (1978–2021) dynamics of SO_4_ and NO_3_ concentrations from the two LTER and ICP WATERS lake sites (PAI and PAS). The SO_4_ and NO_3_ long-term data were also provided from a pristine mountain stream as a further case study.

## Results

### Atmospheric deposition

**Fig. 2:**
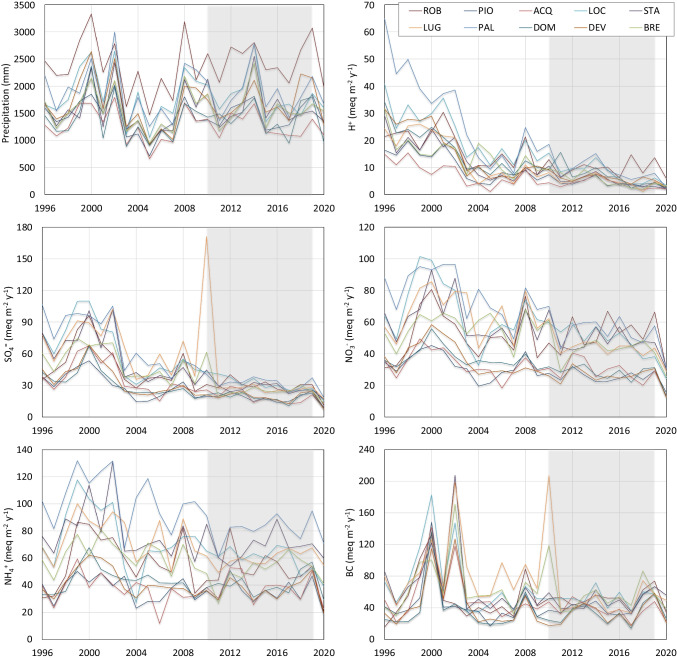
Annual precipitation amount and average deposition of acidity, SO_4_, NO_3_, NH_4_, and base cations (BC) during 1996–2020 at 10 sites (those with the longest data series). The peaks of SO_4_ and BC deposition in Lugano in 2010 were due to the airborne volcanic plume of the Eyjafjallajokull eruption in Iceland (Steingruber, 2015). Acronyms as in Table 1. Gray area: reference period (2010–2019)

In 2020, mean ± SD precipitation amount at the study sites was 1476 ± 288 mm, from 964 (at DOM) to 1981 mm (at ROB, the highest site) (Fig. 1; Table 1). The 2020 precipitations were below 1732 ± 322 mm, the average ± SD precipitations in the reference period (2010–2019), but still within the usual range of variation for each site (Fig. 2; Table 2). In 2020, the highest concentrations of sulfur and nitrogen compounds were recorded in the precipitation samples collected at the southern sites (e.g., PAL, LUG, STA) while the lowest at the northern, high-altitude sites (e.g., DEV, ROB, PIO). Ammonium was the dominant form of N, with concentrations ranging between 10 and 44 µeq L^−1^. The corresponding range for NO_3_ and SO_4_ concentrations were 10–22 µeq L^−1^ and 12–25 µeq L^−1^ respectively.

**Table 2:** Precipitation amount and average annual concentrations of the main chemical variables in atmospheric deposition in 2020 and in the reference period 2010–2019 (± standard deviation). Acronyms as in Table 1. BC, base cations. Ion concentrations in µeq L^−1^. Precipitation volume is not measured at BRE and values of the nearby station LUG were used to calculate deposition. BC data are not available for the PIO site

Site	mm 2020	mm 2010–2019	SO_4_ 2020	SO_4_ 2010–2019	NO_3_ 2020	NO_3_ 2010–2019	NH_4_ 2020	NH_4_ 2010–2019	BC 2020	BC 2010–2019
ACQ	1097	1286 ± 215	8	15 ± 4	12	23 ± 4	21	31 ± 7	20	30 ± 6
BIG	1558	1673 ± 330	9	14 ± 2	15	23 ± 3	19	27 ± 5	29	27 ± 6
BRE	-	-	8	18 ± 6	17	28 ± 5	27	33 ± 8	25	37 ± 13
LOC	1599	1816 ± 402	10	18 ± 4	15	28 ± 5	25	35 ± 7	24	30 ± 6
LUG	1543	1616 ± 357	11	17 ± 3	19	29 ± 4	36	39 ± 7	32	30 ± 9
PIO	1306	1492 ± 249	7	13 ± 2	12	18 ± 3	14	25 ± 6	-	-
ROB	1981	2523 ± 325	6	12 ± 2	15	21 ± 4	10	21 ± 5	17	21 ± 3
SON	1800	2052 ± 288	7	14 ± 3	13	23 ± 4	20	33 ± 7	18	29 ± 6
STA	1350	1646 ± 402	12	18 ± 3	22	31 ± 4	44	44 ± 7	41	30 ± 6
PAL	1669	1891 ± 411	11	18 ± 3	18	30 ± 4	43	45 ± 8	25	26 ± 6
DOM	964	1444 ± 283	8	13 ± 2	13	20 ± 3	30	29 ± 5	27	23 ± 7
DEV	1297	1734 ± 319	6	12 ± 2	9	16 ± 3	15	22 ± 5	16	19 ± 6
All sites	1476	1732 ± 443	9	15 ± 4	15	24 ± 6	25	32 ± 9	25	28 ± 8

Average annual concentrations of SO_4_, NO_3_, and NH_4_ were lower in 2020 with respect to the previous decade at all the sites (except for NH_4_ at STA and DOM). In most cases, the 2020 values marked the minimum value never before reported. No clear pattern emerged for base cations (BC), with some sites having lower and other sites higher concentrations in 2020 (Table 2).

When considering deposition, acidity sharply decreased until around 2005 (Fig. 2), and then the values stabilized between 5 and 10 meq m^−2^ year^−1^, according to the site. In 2020, the average deposition of acidity (H^+^) was the minimum value ever recorded at 8 out of 12 sites. SO_4_ deposition also decreased steeply at all sites until the early 2000s; then, its decrease slowed considerably in the following years, reaching values mostly between 20 and 40 meq m^−2^ year^−1^. In 2020, all the 12 sites recorded the minimum SO_4_ deposition ever recorded. Similar to SO_4_, also NO_3_ reached its minimum values in 2020: however, the effect was much more evident than for SO_4_, with several sites showing concentrations and deposition in 2020 almost halved with respect to the average values of the reference period (Table 2). As an example, NO_3_ deposition at the southern, low-altitude sites PAL (Italy) and LOC (Switzerland) was, respectively, 31 and 24 meq m^−2^ year^−1^ in 2020 and 56 and 50 meq m^−2^ year^−1^ on average, in 2010–2019. At the same time, in the northern, high-altitude sites DEV (Italy) and ROB (Switzerland), NO_3_ deposition was, respectively, 12 and 30 meq m^−2^ year^−1^ in 2020 and 27 and 54 meq m^−2^ year^−1^ on average 2010–2019. Lower deposition in 2020 was also observed for NH_4_ but at a lesser extent than NO_3_ and only at the northernmost sites (Fig. 2). No evident trend occurred in BC deposition, and 2020 values were like those of the previous years (Fig. 2).

**Fig. 3:**
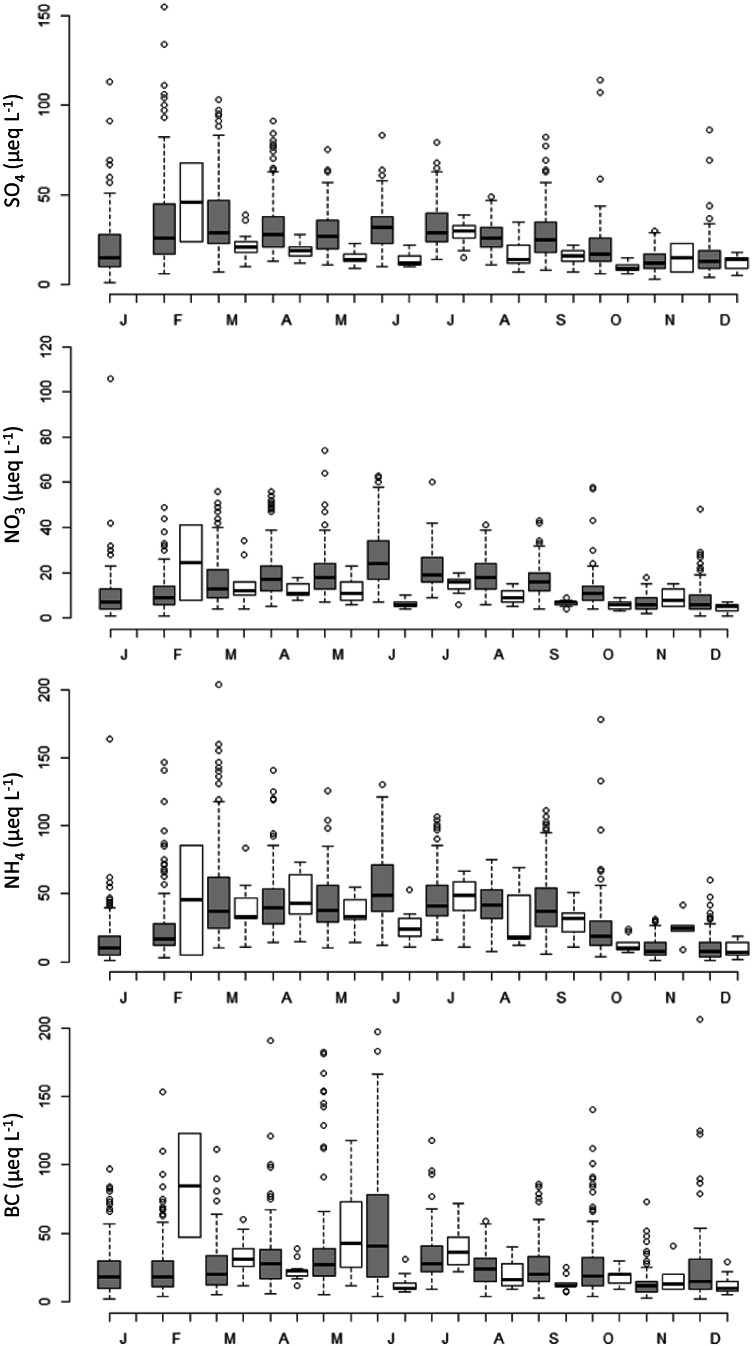
Boxplot comparing the monthly SO_4_, NO_3_, NH_4_, and BC concentrations in precipitations in 2010–2019 (gray boxes) and 2020 (white boxes, including the COVID-19 lockdown period in Italy, from March 2020) in 12 atmospheric deposition sampling sites. Boxes, median, upper and lower quartiles; whiskers, ± 1.5 × interquartile range (IQR) or minimum/maximum values whichever is closest to the median

The results of LMEs confirmed a significant decrease of SO_4_ and NO_3_ during the lockdown, while no significant differences were found for NH_4_ and BC before and after the lockdown period (Table 4). As expected, precipitation volume had a significant dilution effect on the concentrations of all the considered compounds; the interaction between precipitation amount and COVID-19 significantly affected SO_4_ and NH_4_ concentrations indicating that the dilution effect mentioned above was stronger during the lockdown than in the previous period, i.e., with the same precipitation amount, solute concentrations were lower during the lockdown. The effects of the lockdown on the concentration and dilution of NO_3_, SO_4_, and NH_4_ are also clearly reflected in the significant lower deposition rates recorded during the lockdown (Table 3, part b). In Fig. 3, we provide a graphic comparison of the NO_3_, SO_4_, NH_4_, and BC concentrations in 2010–2019 vs. 2020, where clearly lower NO_3_ and SO_4_ concentrations can be observed during the lockdown period, in particular between March and October, while differences leveled off at the end of the year.

**Table 3:** Results of linear mixed effects models (LMEs) testing: a, the effects of COVID (binary variable, encoding for pre- and post-COVID-19 lockdown samplings), precipitation volume (PREC), and their interaction on the monthly log-transformed concentrations of NO_3_, SO_4_, NH_4_, and basic cations (BC) at 12 sampling sites; b, the effects of on COVID SO_4_, NO_3_, NH_4_, and BC deposition at the same sampling sites. Significant *p* levels are in bold

Dependent variable	Covariates	beta	SE	Df	t	p
a Concentration						
log(NO_3_)	(Intercept)	3.133	0.12	22.11	26.46	**0.000**
	COVID	− 0.357	0.08	9.18	− 4.69	**0.001**
	PREC	− 0.153	0.01	1615.00	− 12.28	**0.000**
	COVIDxPREC	− 0.002	0.04	1602.00	− 0.05	0.958
log(SO_4_)	(Intercept)	2.534	0.15	17.82	16.85	**0.000**
	COVID	− 0.406	0.11	10.05	− 3.60	**0.005**
	PREC	− 0.088	0.02	1620.32	− 5.85	**0.000**
	COVIDxPREC	− 0.098	0.05	1599.28	− 1.99	**0.047**
log(NH_4_)	(Intercept)	3.175	0.22	16.27	14.30	**0.000**
	COVID	− 0.144	0.11	13.15	− 1.30	0.216
	PREC	− 0.152	0.02	1614.67	− 9.10	**0.000**
	COVIDxPREC	− 0.123	0.06	1602.16	− 2.24	**0.025**
log(BC)	(Intercept)	3.100	0.12	24.47	25.88	**0.000**
	COVID	− 0.108	0.13	13.51	− 0.80	0.436
	PREC	− 0.107	0.02	1623.76	− 5.26	**0.000**
	COVIDxPREC	− 0.066	0.07	1608.66	− 0.99	0.324
b Deposition						
log(NO_3__dep + 1)	(Intercept)	1.328	0.11	19.17	12.19	**0.000**
	COVID	− 0.346	0.07	10.75	− 4.98	**0.000**
log(SO_4__dep + 1)	(Intercept)	0.991	0.11	14.98	9.04	**0.000**
	COVID	− 0.362	0.07	11.92	− 5.19	**0.000**
log(NH_4__dep + 1)	(Intercept)	1.422	0.17	14.18	8.40	**0.000**
	COVID	− 0.251	0.07	11.66	− 3.50	**0.005**
log(BC_dep + 1)	(Intercept)	1.330	0.12	18.88	11.40	**0.000**
	COVID	− 0.132	0.12	12.38	− 1.09	0.297

The relative decreases of concentration and deposition in 2020, calculated with respect to the previous decade, confirmed a marked decrease of both SO_4_ and NO_3_ concentrations (on average − 46% and − 38%, respectively), despite differences among sites (Table 4). The highest relative changes of NH_4_ concentrations were detected at the high-altitude northeastern sites (− 40/ − 50% at ROB and PIO), while limited or no changes occurred at the southern sites (PAL, DOM, STA, LUG). Base cations showed striking spatial differences, increasing at some sites (e.g., STA, DOM) and decreasing at others. Deposition data showed the same pattern, with even higher relative changes due to the fact the precipitation amount was 5–33% lower in 2020 than in 2010–2020. Due to the uneven spatial distribution of precipitation, higher differences among sites can be also noticed for deposition. However, average decrease of SO_4_ and NO_3_ was quite high (− 54 and − 46%, respectively), while NH_4_ and BC deposition changed at a lesser extent (− 34 and − 25%) and mostly at the higher altitude sites (Table 4).

**Table 4:** % decrease (Δ) of SO_4_, NO_3_, NH_4_, and BC deposition in 2020 with respect to the reference period (2010–2019) at the study sites. Acronyms as in Table 1

	Concentration				Deposition			
Site	ΔSO_4_	ΔNO_3_	ΔNH_4_	ΔBC	ΔSO_4_	ΔNO_3_	ΔNH_4_	ΔBC
ACQ	− 50	− 48	− 34	− 32	− 57	− 55	− 43	− 43
BIG	− 34	− 35	− 31	7	− 38	− 38	− 35	− 2
BRE	− 53	− 41	− 18	− 32	− 54	− 43	− 20	− 35
LOC	− 47	− 46	− 30	− 19	− 52	− 52	− 36	− 27
LUG	− 56	− 35	− 8	− 15	− 59	− 36	− 9	− 20
PIO	− 48	− 34	− 43	-	− 54	− 41	− 50	-
ROB	− 54	− 29	− 52	− 19	− 64	− 44	− 62	− 36
SON	− 52	− 45	− 40	− 39	− 58	− 51	− 46	− 47
STA	− 31	− 27	2	38	− 42	− 39	− 14	14
PAL	− 39	− 39	− 5	− 7	− 45	− 45	− 14	− 23
DOM	− 39	− 35	4	19	− 59	− 56	− 29	− 23
DEV	− 50	− 41	− 34	− 15	− 62	− 54	− 49	− 37
Average	− 46	− 38	− 24	− 10	− 54	− 46	− 34	− 25

To provide a graphic representation of how the lockdown affected precipitation chemistry and deposition, we considered the data of monthly precipitation amount and average concentrations and depositions of SO_4_ NO_3_, NH_4_, and BC at three selected sites placed along a latitudinal gradient and therefore representative of an aggrading level of pollutant deposition (PAL, LOC, ROB). The 2020 data were compared with the reference period (2010–2019) (Fig. 1S). The three sites showed quite different patterns of S and N compound concentrations during the year; however, a general tendency towards lower concentrations since April can be seen at all the sites, especially as regards SO_4_ and NO_3_. NH_4_ concentrations were steadily between 5 and 15 µeq L^−1^ at ROB (except for September), markedly lower than the reference values for this site; they were instead close or above the reference values in summer months at PAL and LOC, particularly in July. The most evident effect of the lockdown can be seen for NO_3_ which reached very low values, especially between April and June, when concentrations at the most polluted sites (PAL, LOC) were comparable to those at the remote site of ROB (10–20 µeq L^−1^). Deposition in 2020 showed a similar pattern: SO_4_ and NO_3_ deposition remained below the reference values for most of the year at the three sites, with some exceptions (e.g., SO_4_ in May at PAL, NO_3_ in August at PAL and LOC, both variable in October at LOC and ROB) in correspondence of quite high precipitation volume. NH_4_ deposition was similar or even higher than the reference values at PAL, while it was steadily below at the mountain site ROB (Fig. 1S).

### Freshwaters

**Fig. 4:**
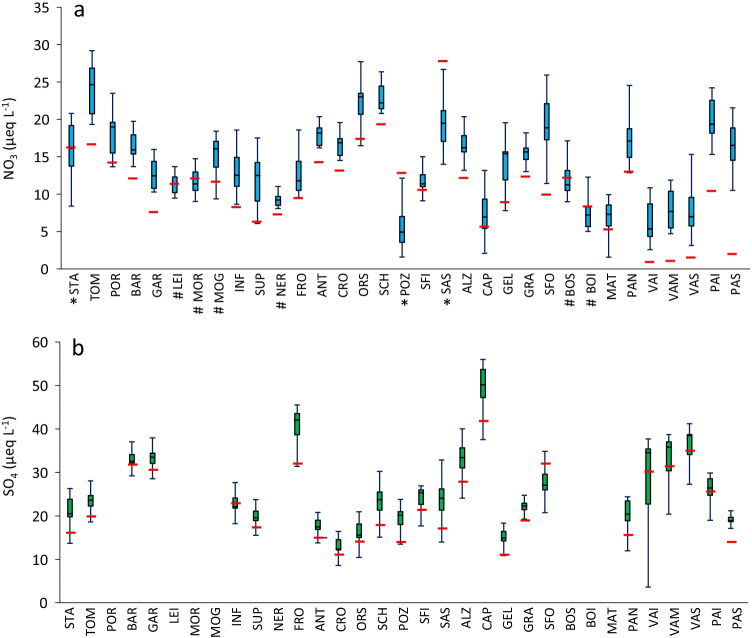
Boxplots of NO_3_ (**a**) and SO_4_ (**b**) concentrations based on data of the period 2010–2019 compared with the 2020 values (red dashes) in 33 high-altitude lakes. *, shallow lakes (< 10 m depth) with more than 25% of vegetation cover in the catchment (see Table 1S). #, lakes with glaciers/rock glaciers in the catchment. Lakes with geological source of SO_4_ were excluded from the comparison in (**b**)

The concentrations of SO_4_ and NO_3_ measured in 2020 in the 33 survey lakes were compared with the data available for the same lakes in the period 2010–2019 (Fig. 4). NO_3_ concentration in 2020 was below the median value of the reference period in most lakes (79%), marking a new minimum recorded value in 18 lakes (Fig. 4a). The decrease of concentrations in 2020 with respect to the previous decade (ΔNO_3_) varied from 15 up to 85% in some lakes (e.g., VAI, VAM, VAS, PAS), where concentrations measured in 2020 were almost negligible (1–2 µeq L^−1^).

When considering SO_4_, the difference between 2020 and the reference period was less evident (Fig. 4b). Some lakes (i.e., BOI, BOS, LEI, MAT, MOG, MOR, NER, POR) with rock glaciers in the catchment or with geological sources of SO_4_ (e.g., S-bearing minerals like gypsum) have much higher concentrations with respect the other lakes (100–250 µeq L^−1^), and such values seem little influenced by the observed decreased depositions following the lockdown. Among the remaining lakes, most of them still showed lower concentrations in 2020 than in 2010–2019, with a relative change (ΔSO_4_) mostly between 10 and 15%.

LME results confirm that the COVID-19 lockdown had a significant and negative effect on both SO_4_ and NO_3_; in addition, when considered the effects of other relevant environmental variables, LMEs highlight the positive effect of the ratio between lake catchment and lake area (BA) on NO_3_ concentration, the positive effect of the presence of glaciers and rock glaciers on the SO_4_ concentration, and the different response of lakes with or without S-bearing minerals in their catchments to the reduced SO_4_ deposition following the lockdown, as indicated by the significant interactive effect between COVID-19 and the presence/absence of S-bearing rocks (Table 5).

**Table 5:** Results of linear mixed effects models (LMEs) testing the effects of COVID (binary variable, encoding for pre- and post-COVID-19 lockdown samplings) on the log-transformed SO_4_ and NO_3_ concentrations in 33 high-altitude lakes. COVID, lake elevation, lake area, catchment area–lake area ratio (BA), percent vegetation cover in the catchment (Vegetation), presence absence of glaciers and rock glaciers in the catchment (Glaciers), and the interaction between COVID and the presence absence of sulfur-bearing rocks (COVIDxS-bearing rocks) were added to the models as covariates; lake identity and the year of sampling were added as additive random effects. Significant *p* levels are in bold

Dependent variable	Covariates	Beta	SE	df	*t*	*p*
Log (NO_3_)	(Intercept)	4.911	1.35	26.90	3.65	**0.001**
	COVID	− 0.378	0.13	8.57	− 2.88	**0.019**
	Elevation	− 0.001	0.00	26.96	− 1.61	0.120
	Area	0.010	0.02	27.33	0.50	0.621
	BA	− 0.004	0.00	35.50	− 2.58	**0.014**
	Vegetation	− 0.008	0.00	27.10	− 1.81	0.082
	Glaciers	− 0.254	0.21	27.31	− 1.19	0.244
Log (SO_4_)	(Intercept)	2.723	1.63	23.57	1.67	0.107
	COVID	− 0.167	0.04	10.30	− 3.98	**0.002**
	S-bearing rocks	0.147	0.11	270.80	1.29	0.199
	Elevation	0.000	0.00	23.73	0.39	0.701
	Area	− 0.036	0.02	24.43	− 1.55	0.134
	BA	0.000	0.00	53.52	0.23	0.816
	Vegetation	0.005	0.01	23.85	0.87	0.393
	Glaciers	1.160	0.26	26.22	4.46	**0.000**
	COVIDxS-bearing rocks	0.178	0.05	291.80	3.32	**0.001**

**Fig. 5:**
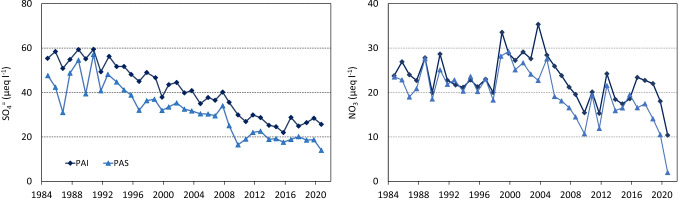
Long-term trends of annual data (autumn sampling) of NO_3_ and SO_4_ concentrations in the LTER and ICP WATERS sites PAI and PAS

A focus on the long-term trends of SO_4_ and NO_3_ is provided for the LTER and ICP WATERS sites PAI and PAS, for which continuous data exist since the early 1980s (Fig. 5). These two lakes, particularly the upper lake PAS, experienced acidification in the 1980s due to high deposition of S and N compounds. Starting from the 1990s SO_4_ concentrations showed an evident and almost regular decreasing trend in response to the decreasing atmospheric input, while NO_3_ had a more irregular pattern, with a slight tendency towards lower values between 2005 and 2010. For SO_4_ a slightly lower value was measured in 2020 in PAS only (14 µeq L^−1^), but it was quite similar to those of the previous years (around 20 µeq L^−1^). On the other hand, NO_3_ in 2020 reached the lowest recorded values from the beginning of the monitoring in both lakes (10 and 2 µeq L^−1^ in PAI and PAS, respectively). We assessed the weather conditions prior to the lake sampling in 2020 by considering the data from the Automatic Weather Station (AWS) located at lake PAS, covering the period 2001–2020 (Fig. 2S). On average, the summer of 2020 (June to August) was drier than usual, with 380 mm of precipitation with respect to 520 mm as long-term average. Minimum and maximum air temperature in the same months, corresponding to the growing season, was slightly higher and lower, respectively, than the long-term average (6.84 and 11.95 °C in 2020 with respect to 6.57 and 12.86 °C). However, none of the above differences were significant (Kruskal–Wallis test *p* = 0.38, 0.07 and 0.20 for precipitation, minimum and maximum air temperature, respectively).

**Fig. 6:**
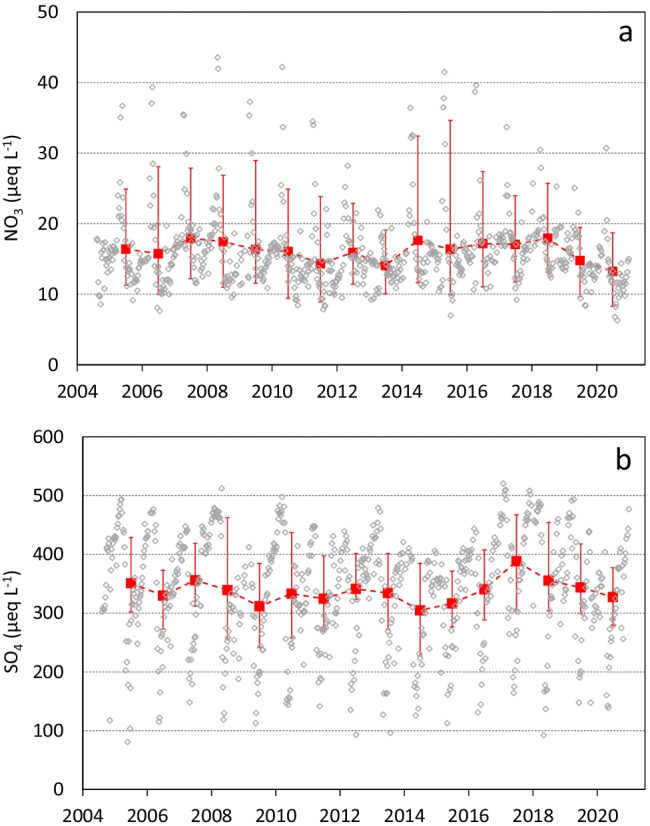
Long-term trends of weekly concentrations of NO_3_ (**a**) and SO_4_ (**b**) in Rio Devero stream. Red squares and lines: annual average values with interquartile range

A further example of the effects of COVID-19 lockdown on surface water chemistry was provided for a stream site, subject to high-frequency monitoring (weekly data) (Fig. 6). Both SO_4_ and NO_3_ concentrations were characterized by a distinct seasonal pattern: SO_4_ usually peaks in winter, then decreases at snowmelt, and reaches the minimum values in May. NO_3_ showed a different seasonal pattern, with a peaking in April to May, during snowmelt, and a minimum in late summer (August to September). In 2020, the seasonal pattern was the same, with a NO_3_ peak in April (31 µeq L^−1^) followed by a decrease reaching very low minimum values in July (6–7 µeq L^−1^) (Fig. 6a). The average NO_3_ concentration in 2020 was 13 µeq L^−1^, the lowest of the entire record, likely due to the very low summer concentrations (e.g., 10 µeq L^−1^ as the average value of July to September 2020, with respect to 14 µeq L^−1^ in 2010–2019). On the contrary, SO_4_ did not change in 2020, with an average annual value (327 µeq L^−1^) very close to the 2010–2019 average (338 µeq L^−1^) (Fig. 6b).

## Discussion

A detailed analysis of the long-term trends in the chemistry of deposition in Lake Maggiore area in relation to changing emission was presented in Rogora et al. (2016), showing that major changes occurred before the 2000s followed by a period of relative stability. The data presented here show that this relative stability continued in the period 2010–2019, which was used as a reference period to test the effects of the COVID-19 lockdown on deposition and freshwater chemistry. Consistently with our first study hypothesis, a widespread decrease in the SO_4_ and NO_3_ precipitation concentration and deposition occurred as a likely effect of the lockdown (on average − 54 and − 46%, respectively, with respect to 2010–2019). The effect seemed more apparent just after March (i.e., between April and June, when restrictions were particularly severe). NH_4_ deposition decreased in 2020 (− 34%) in particular at the northern and highest sites; according to our results, this reduction was attributable to the combined effect of the relatively low precipitations in 2020 and of a stronger dilution effect of precipitation volume in 2020 than in the previous period. The observed decrease in NH_4_ was likely due to the reduction in (NH_4_)_2_SO_4_, which resulted in reduced long-range transport of NH_4_ (ApSimon et al. 1994): this may explain the higher reduction rates observed at the northernmost sites.

Base cations showed an uneven spatial distribution, not affected by the COVID-19 lockdown and likely depending on local factors and/or airborne dust. For instance, Saharan dust events, although rare, have a strong impact on precipitation chemistry in the study area (Rogora et al. 2016). In 2020, two of these events occurred, one in late March and the other in May, causing higher than usual SO_4_ (e.g., PAL in March) and BC concentrations (e.g., LOC in May) (Fig. 1S).

Concentration and depositions of SO_4_ and NO_3_ in 2020 did not decrease uniformly in the study area. A decreasing south-north gradient in the deposition of acidity and S and N compounds has already been described for the study area, due to the location of the major emission sources south of Lake Maggiore watershed (e.g., the metropolitan area of Milan and the whole Po Plain) (Rogora et al. 2016). The same inverse relationship has been found in Southern Switzerland, where sulfur, nitrogen, and potential acidity deposition is much higher at low latitudes and altitudes (Steingruber 2015), confirming the importance of transboundary air pollution originating from the Po Plain in Italy. These gradients reflect the different responses of precipitation chemistry and deposition to the lockdown, with the sharpest decrease of SO_4_ and NO_3_ deposition occurring at the most impacted, southern, and low-altitude sites. On the other hand, the decrease of NH_4_ deposition in 2020 after the lockdown was less evident in the southern part of the area, where both concentrations and depositions remained quite high (25–45 µeq L^−1^ and 40–70 meq m^−2^ y^−1^) and confirmed NH_4_ as the dominant N form in the deposition of inorganic N (60–70% of the total). NH_4_ has indeed become more and more important as an atmospheric source of N (Rogora et al. 2016), because of the limited change that occurred in the emissions of NH_3_ with respect to those of SO_2_ and NO_x_: since 1990, emissions of SO_2_, NO_x_, and NH_3_ in Italy decreased by 90%, 58%, and 14%, respectively (Romano et al. 2014); similarly, in Switzerland, the decreases within 1980–2012 were 90%, 59%, and 25% (Heldstab et al. 2014).

The observed decrease in NO_3_ deposition in the post-lockdown period paralleled that described for NO_2_ air concentrations in Northern Italy and Switzerland (Ciarelli et al. 2021; Grange et al. 2020). The air quality monitoring data from the Po basin area indicated a 40% decrease of NO_x_ emissions (Deserti et al. 2020), mainly attributable to COVID-19 restrictions to mobility. Indeed, the overall transport sector contributes to 61% of the total NO_x_ emissions in Italy (50% by road and 11% by other transport) (EEA 2014). NH_3_ emissions are instead dominated by agricultural sources (95% of the total) (EEA 2014). We interpret the lack of a coherent response in NH_4_ deposition as a direct consequence of the fact that no reductions have been observed for NH_3_ emissions and air concentrations, as agricultural activities have not stopped during the lockdown (Deserti et al. 2020; Lovarelli et al. 2020). Deposition data also showed a general but small decrease of SO_4_ loads during the lockdown. This is likely due to the fact that SO_2_ emissions underwent little changes because of the lockdown (Guevara et al. 2021). Indeed, SO_2_ emissions are related to transport to a lesser extent than N depositions (i.e., 16 vs. 61% of total emissions in northern Italy) and are mainly related to domestic energy use and industrial processes (84%) which continued during the lockdown. Furthermore, SO_2_ emissions were already low before the lockdown thanks to international protocols and measures for emission reductions, making the potential effects of lockdown restrictions less likely to produce further major reductions.

The reductions of NO_3_ and SO_4_ in the high mountain lakes sampled in 2020 are consistent with the decrease of deposition, especially as regards NO_3_, as well as the temporary decrease of NO_3_ concentration occurred in summer 2020 in a high-altitude stream located in a pristine area. Among lakes, there are only a few exceptions to the widespread reduction of NO_3_, corresponding to (i) a few shallow lakes (STA, POZ, SFI, SAS; 4–12 m depth), situated at lower altitudes (1744–1955 m a.s.l.), with abundant vegetation in their catchment, and a large catchment-lake area ratio, favoring the N uptake processes (Table 1S), or (ii) a few lakes with glaciers or large active rock glaciers in their catchment (LEI, MOR, BOS, BOI). In these lakes, NO_3_ concentrations in 2020 were in the same range as measured during the previous decade. Rock glaciers (RG) have been shown to have pronounced effects on lake water chemistry especially on SO_4_ and BC concentrations (Rogora et al. 2020; Steingruber et al. 2021). In addition, Barnes et al. (2014) and Williams et al. (2007) have identified RG as a NO_3_ source though the flushing of microbially active sediments. It is likely that in these lakes, lake/catchment N biogeochemical processes may have masked the effect of decreased N deposition. In particular, in autumn, the period of lake water sampling, mineralization of organic N in sediments may constitute an important NO_3_ source in shallow low-altitude lakes through sediment resuspension and in lakes with RG through flushes of N-rich sediments from the RG to the lakes.

Extremely low NO_3_ concentrations were measured in 2020 in some of the lakes, including the LTER sites PAS and PAI (Fig. 5). Weather conditions before the sampling may affect water chemistry; in particular, warmer temperatures may promote higher N uptake both in catchment soils and in the water, possibly decreasing N concentrations (Oleksy et al. 2020). However, air temperature before the lake sampling in 2020 was not significantly different from the average data of the previous years (Fig. 2S).

Clear but distinct seasonal patterns characterized SO_4_ and NO_3_ concentrations at the stream site, with spring minima of SO_4_ and other solutes (BC, bicarbonate ions), as well as conductivity, caused by the dilution effect of meltwater. SO_4_ in stream water is indeed mainly deriving from the weathering of S-bearing minerals in the draining catchment. The different behaviors of NO_3_, with peaks at snowmelt, when discharge increases, confirm atmospheric deposition (accumulated as snow) as its main source (Mosello et al. 2002). This seasonal pattern of NO_3_ in 2020 also confirmed the effect of lower N deposition, with particularly pronounced minima (Fig. 6a).

A role of meteo-climatic factors in the N dynamics of the study sites cannot be excluded: air temperature has progressively increased in the study area (Rogora et al. 2013; 2020), possibly enhancing nutrient use and primary production in lakes. Nonetheless, on our opinion, the pronounced decrease of NO_3_ occurred in 2020 cannot be ascribed solely to meteorological drivers but must be necessarily related to the change in N deposition.

A weaker response of freshwater sites to changing deposition was observed for SO_4_: this may be because the study lakes have experienced a sharp decrease of SO_4_ concentrations in the 1980s and 1990s, as an effect of decreasing deposition (Rogora et al. 2013), and they have now reached quite stable and low concentrations which are probably close to the pre-acidification values. However, the effects of the lockdown still significantly reduced SO_4_ concentration at least in the lakes without a known source of SO_4_ in the catchment (RG outflowing waters, S-bearing minerals in the bedrock), where the abundant input of SO_4_ from the catchment masks the effect of reduced deposition.

## Conclusion

The results obtained from this study confirmed the role of atmospheric deposition as a vehicle of air pollutants deriving from anthropogenic activities to terrestrial and aquatic ecosystems. Monitoring data on rain chemistry from Northern Italy and Southern Switzerland showed rapid changes in response to the drop of emissions occurred in 2020, especially of NOx, due to mobility restrictions imposed by the COVID-19 lockdown. On the other hand, less significant changes were observed in the deposition of NH_4_, due to the lack of evident effects of the lockdown on NH_3_ emissions, strictly related to agricultural and zootechnical activities. Freshwater chemistry at long-term monitoring sites showed a coherent and rapid response to changing deposition, mainly in the form of decreasing NO_3_ concentrations.

The rapid chemical recovery observed at lake and stream sites in response to the sharp decrease of deposition put in evidence the high resilience potential of the studied natural ecosystems placed in pristine regions. It also demonstrated the great potential of emission reduction policy in producing further substantial ameliorations of the water quality at sensitive sites: the restrictions imposed under COVID-19 demonstrated that ecosystem quality can be still improved and, in the case of air quality, gets close to pre-industrial condition.

The examples provided in this study highlighted how rapidly high mountain lakes and streams can recover from atmospheric pollution in response to the progressive reduction of S and N oxide emissions that occurred in the last decades or to the sudden and substantial reduction caused by the COVID-19 lockdown. The results also confirmed that small headwater catchments, located in pristine areas or subject to low anthropogenic disturbance, are ideal sites to assess the response of surface waters to changing deposition of S and N compounds and likely to other long-range atmospheric pollutants. This aspect should be considered in the establishment and/or the maintenance of monitoring networks dealing with the effects of air pollution on ecosystems, such as those foreseen according to Art. 9 of the EU NEC Directive.

The transient reduction in the input of atmospheric pollutants and particularly of NO_3_ which occurred in 2020 may be considered as a simulation of further reduction of S and N emission and deposition with respect to the presently achieved reduction and hence represent an interesting case study to investigate the effects of a sharp and rapid emission decrease on atmospheric deposition and on sensitive freshwaters sites. Long-term data from monitoring networks such as LTER and ICP WATERS represent an invaluable asset in such evaluation. The incoming monitoring data will be essential to assess the length of the effects of improved of quality on both deposition and surface water.

## Supplementary Information

Below is the link to the electronic supplementary material.

Supplementary file1 (DOCX 225 KB)

## Data Availability

The datasets generated and analyzed during this study are available from the corresponding author on reasonable request.
