# Supplementary material for: Response of atmospheric deposition and surface water chemistry to the COVID-19 lockdown in an alpine area
Source: Environ Sci Pollut Res Int. 2022 Apr 9;29(41):62312–29. doi: 10.1007/s11356-022-20080-w (PMC8994528; doi:10.1007/s11356-022-20080-w)
Supplement: Supplementary file 1 — Supplementary file1 (DOCX 225 KB) [file 11356_2022_20080_MOESM1_ESM.docx]

| **Pallanza** | **Locarno** | **Robiei** |
| --- | --- | --- |
|  |  |  |
|  |  |  |
|  |  |  |
|  |  |  |
|  |  |  |
|  |  |  |
|  |  |  |
|  |  |  |
|  |  |  |

Fig. 1S – Precipitation amount (mm) and monthly concentrations and depositions of SO_4_, NO_3,_ NH_4_ and BC at three sites along a latitudinal gradient (Fig.1; Tab. 1) in 2020 (blue diamonds) compared with the interquartile distributions (pale blue area: 25^th^ and 75^th^ percentiles; green dotted line: median value) of the same variables in the period 2010-2019. Precipitation was absent or extremely in January 2020 at PAL and LOC. The high concentration of solutes in February 2020 in Locarno was due to the only precipitation event occurred in this month, characterized by a scarce precipitation volume (4.1 mm)

|  |
| --- |

Fig.2S - Minimum and maximum air temperature (monthly average) measured by the weather station at Lake PAS in 2020 compared with the long-term average (2001-2019).

Tab. 1S – Main characteristics of the survey lakes included in the present study.

| **Lake** | **Acronym** | **Lat. N** | **Long. E** | **Altitude** | **Lake area** | **Catchment area** | **Bare rock** | **Brushes and meadows** | **Water (glaciers)** |
| --- | --- | --- | --- | --- | --- | --- | --- | --- | --- |
|  |  | ° ' " | ° ' " | m a.s.l. | ha | ha | % | % | % |
| Starlaresc da Sgiof | STA | 46 16 26 | 08 46 25 | 1875 | 1.1 | 23 | 64 | 32 | 4 |
| Tomè | TOM | 46 21 47 | 08 41 23 | 1692 | 5.8 | 294 | 86 | 9 | 2 |
| Porchieirsc | POR | 46 22 33 | 08 44 39 | 2190 | 1.5 | 43 | 92 | 5 | 3 |
| Barone | BAR | 46 24 07 | 08 45 06 | 2391 | 6.6 | 51 | 83 | 4 | 13 |
| Gardiscio | GAR | 46 45 22 | 08 45 22 | 2580 | 1.1 | 12 | 84 | 7 | 9 |
| Leit | LEI | 46 27 55 | 08 43 17 | 2260 | 2.7 | 52 | 80 | 13 | 7 |
| Morghirolo | MOR | 46 27 03 | 08 43 00 | 2264 | 11.9 | 166 | 81 | 7 | 7 (5) |
| Mognòla | MOG | 46 25 49 | 08 41 19 | 2003 | 5.4 | 197 | 92 | 5 | 3 |
| Laghetto Inferiore | LAI | 46 28 34 | 08 35 34 | 2074 | 5.6 | 182 | 84 | 7 | 7 |
| Laghetto Superiore | LAS | 46 28 34 | 08 35 05 | 2128 | 8.3 | 125 | 88 | 6 | 7 |
| Nero | NER | 46 26 58 | 08 32 22 | 2387 | 12.7 | 72 | 73 | 10 | 17 |
| Froda | FRO | 46 26 24 | 08 33 29 | 2363 | 2 | 67 | 93 | 4 | 3 |
| Antabia | ANT | 46 23 08 | 08 29 32 | 2189 | 6.8 | 82 | 90 | 2 | 9 |
| Crosa | CRO | 46 22 16 | 08 28 60 | 2153 | 16.9 | 194 | 90 | 2 | 9 |
| Orsalia | ORS | 46 20 23 | 08 31 24 | 2143 | 2.6 | 41 | 87 | 7 | 6 |
| Schwarzsee | SCH | 46 20 10 | 08 30 11 | 2315 | 0.3 | 24 | 90 | 8 | 2 |
| Pozzöi | POZ | 46 15 52 | 08 28 17 | 1955 | 1.1 | 33 | 67 | 26 | 4 |
| Sfille | SFI | 46 15 52 | 08 29 46 | 1909 | 2.8 | 63 | 74 | 21 | 5 |
| Sascòla | SAS | 46 17 01 | 08 34 11 | 1740 | 3.2 | 90 | 56 | 40 | 4 |
| Alzasca | ALZ | 46 15 58 | 08 35 05 | 1855 | 10.4 | 110 | 38 | 53 | 9 |
| Capezzone | CAP | 45 56 26 | 08 12 36 | 2100 | 0.9 | 32 | 69 | 29 | 2 |
| Grande | GRA | 46 00 15 | 08 04 44 | 2269 | 0.8 | 90 | 98 | 2 | 1 |
| Sfondato | SFO | 46 00 30 | 08 05 20 | 2422 | 0.5 | 20 | 97 | 0 | 3 |
| Paione Inferiore | PAI | 46 10 09 | 08 11 24 | 2002 | 0.9 | 126 | 90 | 8 | 2 |
| Paione Superiore | PAS | 46 10 26 | 08 11 27 | 2269 | 0.7 | 50 | 93 | 5 | 2 |
| Variola Superiore | VAS | 46 10 48 | 08 12 42 | 2190 | 0.9 | 58 | 92 | 6 | 2 |
| Variola Medio | VAM | 46 10 35 | 08 12 55 | 2130 | 0.7 | 84 | 89 | 8 | 3 |
| Variola Inferiore | VAI | 46 10 30 | 08 13 02 | 2117 | 0.3 | 86 | 89 | 8 | 3 |
| Boden Inferiore | BOI | 46 26 30 | 08 27 01 | 2342 | 5.9 | 91 | 81 | 15 | 4 |
| Boden Superiore | BOS | 46 26 23 | 08 27 04 | 2348 | 2.8 | 30 | 84 | 7 | 9 |
| Gelato | GEL | 46 15 01 | 08 26 30 | 2418 | 0.8 | 15 | 93 | 0 | 7 |
| Matogno | MAT | 46 15 03 | 08 24 05 | 2087 | 3 | 136 | 29 | 69 | 2 |
| Panelatte | PAN | 46 12 10 | 08 27 29 | 2063 | 0.6 | 12 | 52 | 42 | 6 |
